# Phytochemical Characterization and Antioxidant Activity of *Cajanus cajan* Leaf Extracts for Nutraceutical Applications

**DOI:** 10.3390/molecules30081773

**Published:** 2025-04-15

**Authors:** Mariel Monrroy, José Renán García

**Affiliations:** 1 Research Center in Biochemistry and Applied Chemistry, Faculty of Natural and Exact Sciences, Autonomous University of Chiriqui, David P.O. Box 0427, Panama; 2 Department of Chemistry, Faculty of Natural and Exact Sciences, Autonomous University of Chiriqui, David P.O. Box 0427, Panama; 3 National Research System (SNI), National Secretariat of Science, Technology and Innovation (SENACYT), Panama City 0816-02852, Panama

**Keywords:** *Cajanus cajan*, phytochemical characterization, nutraceutical applications

## Abstract

*Cajanus cajan* (guandú) is a widely cultivated leguminous plant in Panama; however, its phytochemical composition remains underexplored. Traditionally used in Asia and Africa for its medicinal properties, the plant’s bioactive compounds responsible for these benefits have not been fully identified. The phytochemical profile and antioxidant capacity of *C. cajan* leaf extracts from Panama were characterized, highlighting their potential applications. Ethanolic extracts obtained via ultrasonication were analyzed through phytochemical screening, confirming the presence of alkaloids, tannins, saponins, and steroids. Spectrophotometric analysis revealed high total phenolic (71 mg g^−1^) and flavonoid (30 mg g^−1^) contents. Antioxidant assays demonstrated significant 2,2′-azino-bis(3-ethylbenzothiazoline-6-sulfonic acid) radical cation (ABTS^+^) inhibition and 2,2-diphenyl-1-picrylhydrazyl (DPPH) radical scavenging activity. Gas chromatography–mass spectrometry (GC-MS) analysis identified 35 bioactive compounds in *C. cajan* leaves for the first time, including lupeol (antimicrobial and antitumor), lupenone (antidiabetic), squalene (antitumor and antioxidant), tocopherol (antioxidant), and β-amyrin (antibacterial and anti-Alzheimer’s). These findings expand the known phytochemical profile of *C. cajan*, supporting its pharmaceutical, nutraceutical, and agro-industrial potential. Moreover, this research provides a foundation for further studies on the plant’s bioactive compounds and their applications in human health and sustainable agriculture.

## 1. Introduction

*Cajanus cajan* (L.) Millsp., commonly known as “guandú”, “gandul”, or “pigeonpea”, is a perennial leguminous plant widely cultivated in tropical and subtropical regions [1]. It is the sixth most produced food legume globally, following common beans (*Phaseolus vulgaris* L.), chickpeas (*Cicer arietinum* L.), field peas (*Pisum sativum* L.), cowpeas (*Vigna unguiculata*), and lentil peas (*Lens culinaris*) [2]. Cultivation of *C. cajan* spans approximately 5.4 million hectares worldwide, yielding over 4 million tons annually, with India accounting for more than 79% of global production [3]. Beyond its role in food security, *C. cajan* is valued for its soil-enriching properties through nitrogen fixation and its high nutritional content, providing essential minerals, B vitamins, and proteins [4].

In addition to its nutritional significance, *C. cajan* has traditionally been used in Asian and African medicine for treating various diseases, such as diabetes, ulcers, skin irritation, hepatitis, measles, jaundice, dysentery, and nervous disorders [1,5]. Different parts of the plant exhibit medicinal properties with widely documented medicinal applications. The leaves alleviate urinary discomfort, chronic joint pain, diabetes, inflammation, ulcers, malaria, epilepsy, colds, bronchitis, poisoning, and genital and skin inflammation. They also possess laxative, coagulant, analgesic, diuretic, and hypocholesterolemic properties [1,6,7]. The flowers are used to alleviate respiratory infections, colds, pneumonia, bronchitis, and menstrual disorders [8]. The seeds function as energy stimulants, poultices, and anti-dizziness remedies [1,9]. Furthermore, the roots are traditionally used as an expectorant, antiparasitic, and treatment for syphilis and poisoning [8]. Additionally, *C. cajan* has been reported to aid in treating gingivitis and stomatitis, stimulate lactation, promote oral hygiene, and exhibit antimicrobial, anti-inflammatory, antioxidant, and anticancer properties [10,11].

Building on the therapeutic properties of *C. cajan*, studies conducted in India, China, and Nigeria have demonstrated the antimicrobial potential of ethanolic and methanolic extracts of *C. cajan* leaves, seeds, and roots. These extracts have shown antimicrobial activity against various microorganisms, including *Staphylococcus aureus*, *Bacillus subtilis*, *Streptococcus* sp., *Salmonella thypi*, *Klebsiella* sp., and *Escherichia coli* [12,13]. Despite these promising findings, the medicinal properties of *C. cajan* remain partially understood, as not all effects have been associated with specific molecules.

The plant, especially its leaves, is a rich source of bioactive compounds, including flavonoids, stilbenes, isoflavones, coumarins, and triterpenes, contributing to its pharmacological potential [6,14]. Among these, phenolic compounds and flavonoids play a crucial role in antioxidant activity by scavenging free radicals and reducing oxidative stress, which is associated with various chronic diseases, including cancer, cardiovascular disorders, and neurodegenerative conditions [15,16,17]. These compounds have demonstrated potent antioxidant, anticancer, and antimicrobial properties that can modulate lipid metabolism and inflammatory responses [5,6,18].

Despite these promising findings, the phytochemical composition of *C. cajan* remains underexplored outside of Asia and Africa. The concentration and profile of its bioactive compounds can vary based on geographic location, soil composition, plant cultivar, and agricultural practices. However, few studies have examined *C. cajan* in Panama and Latin America. To address this gap, the present study aims to chemically characterize *C. cajan* leaf extracts from Panama by determining their phenolic, flavonoid, and anthocyanin content, as well as evaluating their antioxidant activity using 2,2′-azino-bis(3-ethylbenzothiazoline-6-sulfonic acid) (ABTS) and 2,2-diphenyl-1-picrylhydrazyl (DPPH) assays. These findings contribute to the understanding of the plant’s phytochemical profile and its potential for future nutraceutical and biotechnological research.

## 2. Results

### 2.1. Chemical Composition of C. cajan Leaves

**Figure 1:**
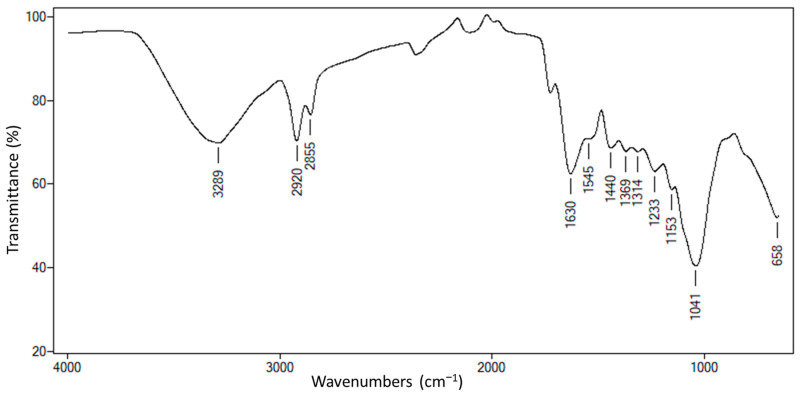
FT-IR spectrum of *Cajanus cajan* (L.) Millsp.

Carbohydrate analysis revealed glucose and mannose in the *C. cajan* leaves, with concentrations of 134 and 56 g kg^−1^, respectively. The Fourier transform infrared (FT-IR) spectrum of *the C. cajan* leaves revealed functional groups characteristic of lignocellulosic materials (Figure 1). The absorption bands at 3289 and 2920 cm^−1^ correspond to the stretching vibrations of the hydroxyl and aliphatic C–H functional groups, respectively, suggesting the presence of carbohydrates or amino acids, and lipids [19]. The band at 1707 cm^−1^ is associated with C=O stretching vibrations of the ester carbonyl or carboxylic acid functional group. Additionally, the –C=O absorption can be assigned to conjugated or nonconjugated, saturated or unsaturated amides, acids, or other systems. Peaks associated with aromatic C stretching were identified at 1545 and 1440 cm^−1^, whereas the peak at 1314 cm^−1^ can be attributed to C–H asymmetric deformation and COO− anion stretching. The bands in the 1440–1201 cm^−1^ range were assigned to C–O stretching and O–H deformation vibrations, which are likely part of the flavonoid structure [19]. Finally, the absorption band at 1024 cm^−1^ corresponds to the C–O stretching of either an ester or an ether, and the band at 752 cm^−1^ is attributed to CH bending.

**Figure 2:**
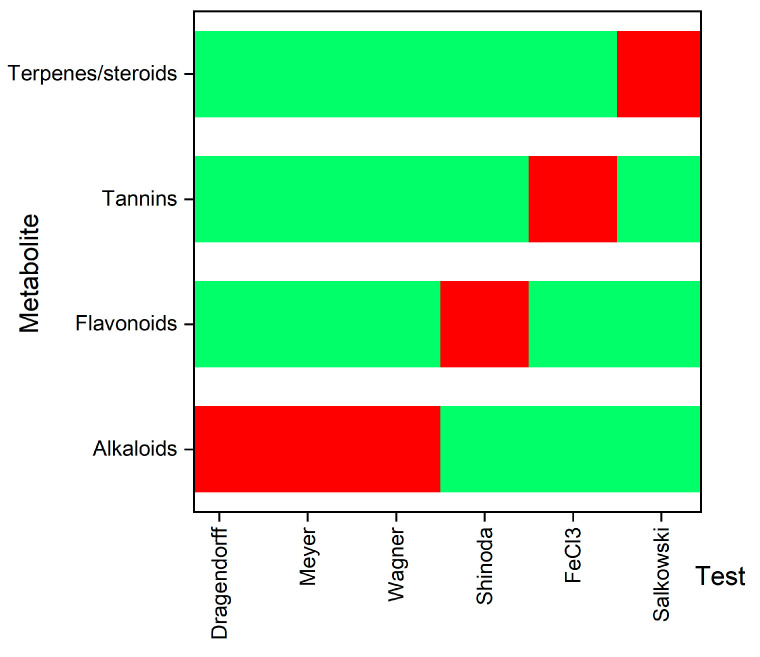
The phytochemical analysis of *C. cajan leaves* shows the presence (red) and absence (green) of phytochemicals in six qualitative tests.

Figure 2 presents the phytochemical analysis results of the *C. cajan* extract, revealing the presence of several bioactive compounds, including alkaloids, flavonoids, tannins, and terpenes/steroids.

### 2.2. Phenolic Compounds, Flavonoids, and Anthocyanin Content

**Figure 3:**
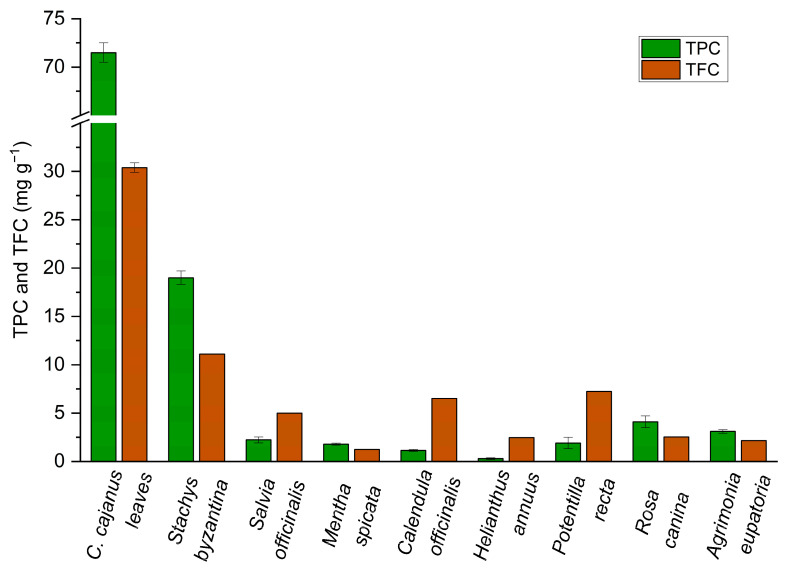
Total phenolic content (TPC) and total flavonoid content (TFC) of *C. cajan* leaves compared to those of other medicinal herbs. The values for medicinal herbs were obtained from Sytar et al. [20]. Values are given as mean ± standard deviation.

Ultrasound-assisted extraction using a 60% hydroethanolic solution yielded a total phenolic content (TPC) of 72 ± 1 mg gallic acid equivalents (GAE) g^−1^, a total flavonoid content (TFC) of 30.4 ± 0.5 mg quercetin equivalents (QE) g^−1^, and a total anthocyanin content (TAC) of 0.41 ± 0.01 mg g^−1^, confirming the bioactive potential of *C. cajan* leaves. A comparison of TPC and TFC values with other medicinal herbs is provided in Figure 3.

### 2.3. C. cajan Leaves Exhibit High Antioxidant Activity According to ABTS and DPPH Assays

**Figure 4:**
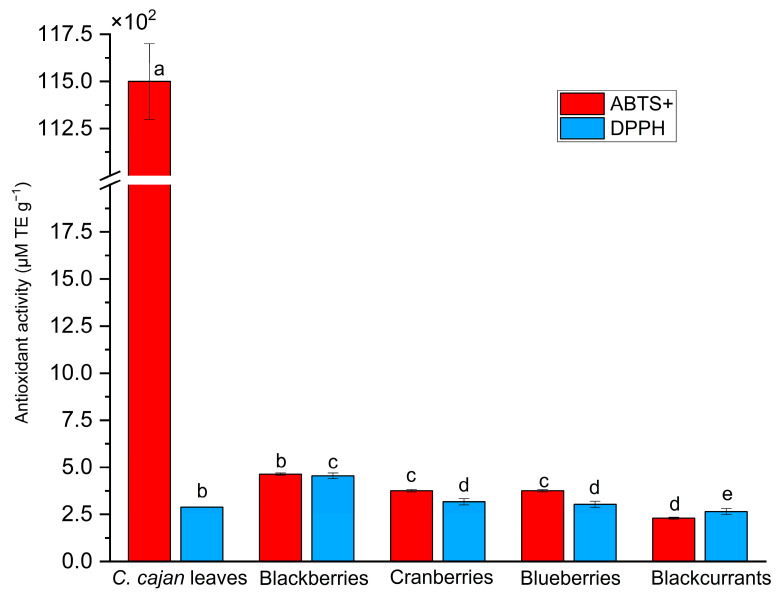
Antioxidant capacity of *C. cajan* leaves compared to common berries, known for their high antioxidant content. Berry values were obtained from Kim et al. [21]. Values are given as mean ± standard deviation (*n* = 6 for *C. cajan* leaves and *n* = 5 for berries). Significant differences between *C. cajan* leaves and berries for each assay (ABTS and DPPH) are indicated by different letters above the bars for each assay (*p* < 0.05).

The antioxidant activity of *C. cajan* leaves was evaluated using ABTS and DPPH assays, which demonstrated that the leaf extract exhibited strong antioxidant activity, with a Trolox equivalent antioxidant capacity (TEAC) value of 11.5 ± 0.2 mM Trolox equivalents (TE) g^−1^ in the ABTS radical cation (ABTS^+^) scavenging assay and 289 ± 1 µM TE g^−1^ in the DPPH assay. Notably, the TEAC values from the ABTS assay were significantly higher (*p* < 0.05) than those from the DPPH assay. The antioxidant capacity of *C. cajan* leaves was then compared to that of commonly consumed berries, such as blackberries, cranberries, blueberries, and blackcurrants (Figure 4).

### 2.4. Identification of Active Compounds by GC-MS

**Figure 5:**
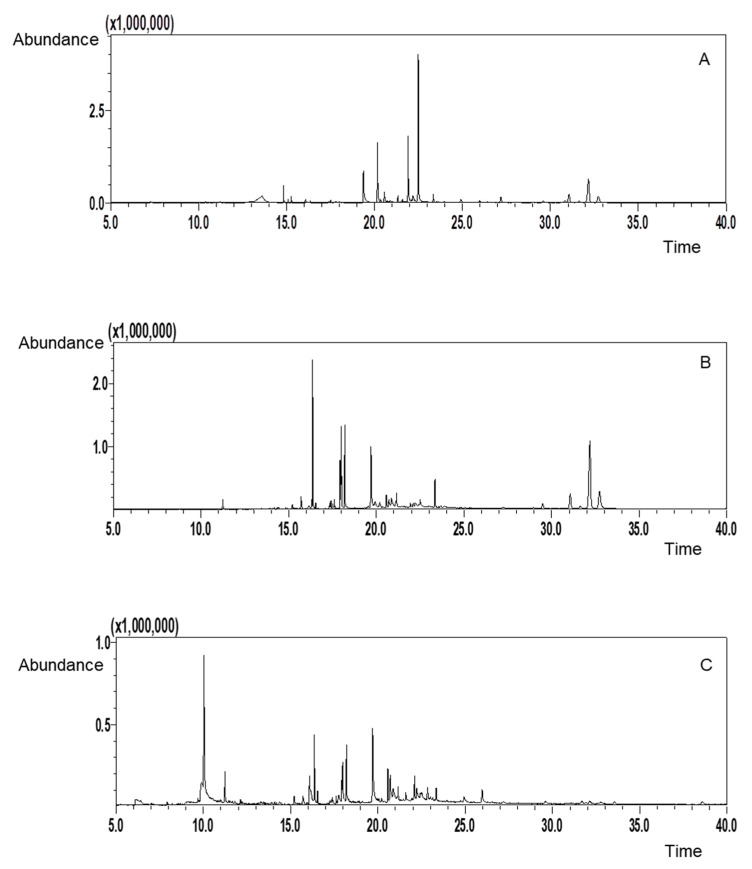
Total ion chromatograms obtained by gas chromatography–mass spectrometry (GC-MS) of *C. cajan* extracts. (**A**) Hydroethanolic crude extract, (**B**) ethanolic extract obtained by liquid–liquid extraction (LLE) with ethyl acetate (EtOAc), and (**C**) ethanolic extract obtained by LLE with hexane. The chromatograms display molecular ion peaks corresponding to the various compounds present in each extract. Differences in the profiles reflect variations in the chemical composition of the extracts based on the extraction solvent used. This analysis highlights the diversity of bioactive compounds in *C. cajan* leaves.

Gas chromatography–mass spectrometry (GC-MS) analysis provides valuable insight into the volatile organic compounds in *C. cajan* leaves, revealing a diverse array of bioactive molecules with potential pharmacological and industrial applications. The total ion chromatograms (Figure 5) depict the signals corresponding to these active compounds, which were categorized based on their elution time, molecular formula, molecular weight, and fragment ions (Table 1). The bioactive compounds were identified by GC-MS analysis with a similarity index of >85%. This comprehensive characterization lays the foundation for further exploration of individual compounds and their respective biological activities.

Among the 44 compounds identified, several have been previously reported in *C. cajan*, including hexadecanoic acid, methyl palmitate, ethyl palmitate, α-guaiene, pinostrobin chalcone, 2,4-di-tert-butylphenol, phytol, α-selinene, and α-himachalene [1,11,18,22,23,24,25]. However, this current study expands the list by identifying 35 new compounds in *C. cajan* leaves, comprising terpenoids (sesquiterpenes, diterpenes, and triterpenes), phenolic compounds, flavonoids, and fatty acids. Notably, several of these compounds have also been reported in various plant species, fungi, and bacteria and have individually been shown to exhibit antioxidant, anti-inflammatory, antimicrobial, and anticancer properties (Table 2).

## 3. Discussion

The measured carbohydrate content of *C. cajan* leaves exceeded the total carbohydrate content of 63 g kg^−1^ reported by Sahu et al. [85] in India but was lower than the 656 g kg^−1^ reported by Yang et al. [86] in Taiwan. Such variations may be attributed to differences in plant variety, geographical origin, and environmental conditions, which influence carbohydrate content. These findings highlight the significant variability in carbohydrate composition across different regions and cultivation practices, underscoring its potential implications for both the nutritional and medicinal uses of *C. cajan.*

The FT-IR spectrum of *C. cajan* also revealed the presence of functional groups, such as polyphenols, pyranose, and fatty acids. Similar spectra have been reported for other plants and lignocellulosic materials, highlighting their structural similarities and the presence of compounds with potential antioxidant and antimicrobial properties [19,87].

The phytochemical composition of *C. cajan* includes alkaloids, flavonoids, tannins, and terpenes/steroids and is consistent with previous findings by Anadebe et al. [24]. Similarly, Sahu et al. [85] and Devi et al. [12] reported the presence of alkaloids, tannins, flavonoids, and saponins but did not identify terpenes. These variations may be attributed to differences in extraction methods, highlighting the influence of extraction techniques on the composition of bioactive compounds in *C. cajan*.

The observed TPC and TFC values confirm the bioactive potential of *C. cajan* leaves and align with or exceed previously reported data. For comparison, Devi et al. [88] reported a TPC of 55 mg GAE g^−1^ and TFC of 36 mg QE g^−1^ using maceration extraction with continuous stirring and ethanol as the solvent. Yang et al. [86] reported TPC values of 13.5 and 7.2 mg GAE g^−1^ using two extraction methods: hot water extraction (decoction) and maceration with 50% ethanol. Also, Yang et al. [86] reported TFC values of 10.4 and 0.2 mg QE g^−1^ by extraction with ethanol and water, respectively, while Aja et al. [89] reported a TFC of 4.2 mg QE g^−1^ and TAC of 0.08 mg g^−1^ by maceration with ethyl acetate (EtOAc). These discrepancies highlight the influence of the extraction techniques on the yield of bioactive compounds. Ultrasonication-assisted extraction, as used in our study, has been reported to enhance cell wall disruption, facilitating the release of phenolic compounds and flavonoids [90], which may explain the higher concentrations observed. However, beyond extraction methods, the concentration and composition of these bioactive compounds are subject to environmental and agronomic factors. Differences in soil composition, climatic conditions, geographic location, and plant cultivar can significantly affect the secondary metabolite profile of *C. cajan* [91,92,93]. For example, studies have demonstrated that phenolic compound accumulation is affected by UV exposure, temperature fluctuations, and soil nutrient availability [91,92,94], further contributing to the discrepancies observed across different studies.

The TPC and TFC values in *C. cajan* leaves were notably higher than those reported for medicinal herbs of the Rosaceae, Asteraceae, and Lamiaceae families (Figure 4). For example, Sytar et al. [20] found TPC values ranging from 1 to 18.6 mg GAE g^−1^ and TFC values between 0.2 and 11.1 mg QE g^−1^ in leaf extracts of these families. These findings suggest that *C. cajan* is a rich source of natural antioxidants, reinforcing its pharmacological properties and traditional medicinal uses.

The presence of phenolic compounds, flavonoids, and anthocyanins in *C. cajan* leaf extract validates its antioxidant and anti-inflammatory potential, positioning it as a promising candidate for nutraceutical and functional food development. Future studies should focus on isolating and characterizing the specific bioactive components responsible for these properties and optimizing extraction techniques to maximize yield and bioactivity.

The antioxidant activity of *C. cajan* leaves was evaluated using ABTS and DPPH assays, both of which operate through the single-electron transfer (SET) mechanism to assess the ability of a substance to donate electrons, neutralizing free radicals and reactive species. These assays provide valuable insights into the plant’s potential to combat oxidative stress [87,95]. Since ABTS and DPPH assays target different radicals, they offer a more comprehensive assessment of antioxidant capacity. The differences between the ABTS and DPPH values in *C. cajan* leaf extract may be attributed to variations in the solubility and reactivity of antioxidants in aqueous versus lipophilic systems. The ABTS assay, which operates in an aqueous system, can better represent the activity of hydrophilic antioxidants, while the DPPH assay primarily interacts with lipophilic compounds, providing a complementary measure of antioxidant capacity.

Interestingly, the ABTS antioxidant capacity of *C. cajan* leaves was significantly higher (*p* < 0.05) than that of commonly consumed berries, such as blackberries, cranberries, blueberries, and blackcurrants, which are widely recognized for their high antioxidant content and thus serve as excellent references in antioxidant studies. These berries exhibit TEAC values of 464 ± 6, 376 ± 6, 261 ± 6, and 230 ± 5 µM TE g^−1^, respectively, in the ABTS assay [21]. Despite *C. cajan* exhibiting lower values (*p* < 0.05) for the DPPH assay compared to the berries, except blackcurrants, its high antioxidant capacity based on the ABTS assay indicates that it is a highly promising natural source of antioxidants.

The remarkable antioxidant activity of *C. cajan* can be attributed to its high phenolic and flavonoid contents, which are well known for their ability to neutralize free radicals and prevent oxidative damage [96], which contribute to the development of various chronic diseases, such as cancer, cardiovascular disease, and neurodegenerative disorders [15,16,17,97]. This positions *C. cajan* as a valuable candidate for the development of functional foods and nutraceuticals. Using *C. cajan* in preventive health strategies could help mitigate the risk of oxidative stress-related diseases, offering a natural plant-based solution for improving human health and well-being.

Further research on the specific bioactive components responsible for these effects and their bioavailability and stability in various formulations is essential to optimize *C. cajan* as a therapeutic agent. Additionally, exploring the interaction of these antioxidants with other bioactive compounds present in plants may provide further insights into their synergistic effects and overall health benefits.

The identification of new compounds in *C. cajan* leaves through GC-MS represents a significant contribution to the understanding of this plant’s bioactive potential. While these compounds have been reported in other plant species, their identification in *C. cajan* highlights a novel aspect of this study. The biological activities associated with these compounds, including antioxidant, anti-inflammatory, antimicrobial, and anticancer properties (Table 2), open new possibilities for the therapeutic application of *C. cajan* leaf extracts. Notably, compounds like α-selinene, oleamide, and levoglucosenone exhibit promising biological activities such as anti-plasmodial, anti-inflammatory, neuroprotective, and bio-based drug discovery potential [29,30,69,70]. Additionally, the identification of triterpenoids like lupeol and lupenone offers exciting prospects for cardiovascular protection, anti-inflammatory action, and anticancer therapy [53,54,55,56]. The discovery of fatty acids, including methyl palmitate and ethyl linoleate, suggests additional therapeutic benefits, including neuroprotective and cardioprotective activity, as well as insecticidal, antibacterial, and anti-inflammatory properties [63,64,67,68]. Interestingly, certain phthalates, such as dibutyl phthalate, di-isobutyl phthalate, and dioctyl phthalate, were also detected. Despite concerns regarding their potential endocrine-disrupting effects, particularly with di-isobutyl phthalate, these compounds offer valuable industrial applications as plasticizers, biosensors, and in other technologies, underscoring their importance in various fields [75,76,77,78,82,83,84]. Additionally, compounds like 1,3-ditert-butylbenzene and 1-pentadecene suggest the potential use of *C. cajan* in natural pest control [71,72]. Furthermore, biofuel-related compounds such as heptadecane reinforce the possibility of utilization in renewable energy research [47,48].

These diverse compounds collectively underscore the untapped therapeutic, agricultural, and industrial potential of *C. cajan* leaf extracts, positioning the plant as a promising candidate for further research. Future studies should focus on isolating, characterizing, and understanding the mechanisms of action of these compounds to optimize their applications in pharmaceuticals, nutraceuticals, and agro-industrial sectors.

While the phytochemical profile of *C. cajan* has been widely studied, particularly in research conducted in India and Africa, data on this species in the American region, including Panama, remain scarce. Environmental factors such as climate, soil composition, and geographical location significantly influence the production and accumulation of bioactive compounds, making it essential to explore *C. cajan* under distinct ecological conditions. In this study, we identified new compounds that had not previously been reported in *C. cajan*, highlighting the potential variability in its chemical composition across geographical regions. These findings contribute to filling the existing knowledge gap and emphasize the need for further investigation into how regional variations may affect the phytochemical diversity and pharmacological properties of *C. cajan.*

## 4. Materials and Methods

### 4.1. Chemicals and Reagents

All the chemicals and reagents used were of analytical grade and were purchased from reputable suppliers. The following chemicals and reagents were used in the experiments: H_2_SO_4_, HCl, KCl, CH_3_COONa, Na_2_CO_3_, NaNO_2_, and ethanol from Ensure; AlCl_3_, FeCl_3_, and NaOH from PanReac AppliChem (Darmstadt, Germany); Folin–Ciocalteu reagent, DPPH, and ABTS from Sigma-Aldrich (Saint Louis, MO, USA); and ethyl acetate, hexane, and chloroform from Merck (Boston, MA, USA). Glucose, mannose, arabinose, and xylose were used as external calibration standards, along with gallic acid, quercetin, and Trolox (Sigma-Aldrich).

### 4.2. Raw Materials

Fresh *C. cajan* leaves were harvested from the New Mexican Township, Alanje District, Chiriqui Province (8°25′12.2″ N, 82°43′42.7″ W). The region has a tropical climate, with average temperatures ranging from a maximum of 34 °C to a minimum of 23 °C. The leaves were harvested during the rainy season and collected from agricultural land at an elevation of 15 m above sea level. The soil was sandy loam with a pH of 5.4 and an organic matter (MO) content of 6.4%. The leaves were dried at 60 °C for 48 h, then milled and stored in dark plastic bags under dry conditions until use. Sample analysis started immediately after collection.

### 4.3. Determination of Carbohydrate Composition and Chemical Characterization

The carbohydrate composition was determined following the methodology of Monrroy et al. [98], with modifications. A 300 mg sample was weighed into a test tube, and 3 mL of 72% (*w*/*w*) H_2_SO_4_ was added. Hydrolysis was performed in a water bath (WD10G11B, Thermo Scientific, Waltham, MA, USA) at 30 °C for 1 h and stirred every 10 min. The acid was then diluted to 3% (*w*/*w*) by adding 79 mL of distilled water, and the mixture was transferred to a 250 mL Erlenmeyer flask and autoclaved (25 X-1, All American, Manitowoc, WI*,* USA) at 121 °C for 40 min. After cooling, the residual material was filtered through a sintered glass filter (No. 4). The concentration of monomeric sugars in the soluble fraction was analyzed using high-performance liquid chromatography (HPLC) (1260 Infinity, Agilent, Santa Clara, CA, USA) with a Hi Plex-H column at 65 °C at a flow rate of 0.4 mL min^−1^ and 8.5 mmol L^−1^ H_2_SO_4_. A refractive index detector (G7162A, Agilent, USA) was used, and glucose, mannose, arabinose, and xylose served as external calibration standards. The carbohydrate analysis was performed in triplicate.

Infrared spectra of the samples were obtained using attenuated total reflectance (ATR). Spectra were recorded between 4000 and 650 cm^−1^ using an FT-IR spectrophotometer (Cary 630, Agilent Technologies, USA) equipped with a deuterated triglycine sulfate detector. The diamond ATR sensor was cleaned with ethyl alcohol before each measurement. The spectral resolution was set to 4 cm^−1^, with 64 scans performed for each spectrum.

The samples were subjected to phytochemical screening by conducting Dragendorff, Wagner, and Mayer tests for alkaloids, the Shinoda test for flavonoids, the ferric chloride test for tannins, and the Salkowski test for sterols, following the methods described by Devi et al. [12] and Khan et al. [99].

### 4.4. Extraction of Bioactive Compounds Using Ultrasound-Assisted Extraction

The extracts were obtained via ultrasound-assisted extraction from hydroethanolic solutions. Specifically, 0.5 g of the dried sample was sonicated in 10 mL of 600 g L^−1^ hydroethanolic solution for 20 min in an ultrasonic bath (S60H, Elma, Singen, Baden-Württemberg, Germany). The resulting extracts were then cooled and filtered.

### 4.5. Phenolic Compounds, Flavonoids, and Anthocyanin Content Evaluation

As described previously, TPC was determined using the Folin–Ciocalteu assay [87]. Different diluted extracts (500 μL) were mixed with 2500 μL of Folin–Ciocalteu reagent (0.2 mol L^−1^) and allowed to stand for 5 min. Then, 2 mL of 75 g kg^−1^ sodium carbonate solution was added, and the mixture was incubated in the dark at 25 °C for 2 h. The absorbance of the solutions was measured at 754 nm using a visible light spectrophotometer (Genesys 10S, Thermo Scientific, USA). A calibration curve was prepared using gallic acid solutions (0–12 µg mL^−1^), and results were expressed as milligrams of GAE per gram of dry peel mass.

TFC was determined spectrophotometrically using the AlCl_3_ colorimetric method [100], with modifications. Briefly, 50 µL of the extract was diluted to 5 mL with distilled water, followed by adding 0.3 mL of 50 g L^−1^ NaNO_2_. After 5 min, 0.3 mL of 100 g L^−1^ of AlCl_3_ was added to the mixture. Shortly thereafter, 1 mol L^−1^ sodium hydroxide NaOH was added, and distilled water was added to bring the total solution volume to 10 mL. After 15 min, the absorbance of the solution was measured at 374 nm using an ultraviolet spectrophotometer (Genesys 10S; Thermo Scientific, USA). A calibration curve was constructed using quercetin solutions (0–15 µg mL^−1^), and the flavonoid content was expressed as milligrams of QE per gram of dry mass.

TAC was estimated using the pH differential method [87]. Extract aliquots were adjusted to pH 1.0 and 4.5 using 25 mM KCl and 0.4 M CH_3_COONa buffer solutions, respectively, and allowed to equilibrate for 20 min. Absorbance was measured at 520 and 700 nm. TAC was calculated based on the difference in absorbances at pH 1.0 and 4.5, using the molecular weight of cyanidin-3-glucoside (449.2 g mol^−1^), the dilution factor (DF), and the molar extinction coefficient of cyanidin-3-glucoside (26,900 L mol^−1^ cm^−1^).

Each analysis was performed in sextuplicate to ensure the reliability and reproducibility of the results.

### 4.6. Determination of Antioxidant Activity Using the ABTS and DPPH Assays

The ABTS assay was performed using the method described by Re et al. [101]. The ABTS radical cation was generated by mixing equal portions of 7 mM ABTS solution and 2.45 mM potassium persulfate solution. The mixture was incubated in the dark at 25 °C for 16 h and diluted with 96% ethanol until its absorbance at 754 nm reached 0.7 ± 0.02. For the assay, 1900 µL of the ABTS•^+^ solution was mixed with 100 µL of the diluted extract and incubated at room temperature in the dark for 10 min. The absorbance of the samples was measured at 754 nm using a visible light spectrophotometer (Genesys 10S, Thermo Scientific, USA). ABTS^•+^ scavenging activity was calculated by comparison with a standard curve of Trolox (40 and 320 mmol L^−1^ Trolox), and the results were expressed as mmol of TE per gram of dry peel mass (mmol TE g^−1^).

The DPPH assay was conducted according to Monrroy et al. [87] and Thaipong et al. [102]. A DPPH radical solution was prepared by dissolving 13 mg DPPH in 100 mL methanol. To achieve an absorbance of 0.70 ± 0.02 at 515 nm, 10 mL of this solution was diluted with 45 mL of methanol. For the assay, 100 µL of the extract was mixed with 2900 µL of the DPPH solution and incubated in the dark at room temperature for 30 min. The absorbance of the sample was measured at 515 nm. DPPH scavenging activity was calculated by comparison with a standard curve of Trolox (40 and 600 mmol L^−1^ Trolox). The results are expressed as mmol of TE per gram of dry peel mass (mmol TE g^−1^).

Both assays were performed in sextuplicate. 

### 4.7. Isolation and Purification of Bioactive Compounds via Liquid–Liquid Extraction (LLE)

The hydroethanolic extract was fractioned using EtOAc and hexane. Initially, the extract was evaporated, and the resulting residue was resuspended in water. The aqueous extract (3 mL) was acidified to a pH of 2 with HCl and subjected to liquid–liquid extraction (LLE) with EtOAc (3 × 3 mL). The EtOAc phase was treated with activated charcoal to absorb the pigments, filtered, dried over anhydrous Na_2_SO_4_, and evaporated. The remaining residue was resuspended in ethanol for GC-MS analysis. The LLE process was then repeated using hexane.

### 4.8. Identification of Bioactive Compounds Using GC-MS

The bioactive compounds obtained through LLE with EtOAc and hexane were analyzed using a 7890A GC system coupled with 5975C MS (Agilent Technologies, USA) fitted with an HP-5MS column (30 m in length × 250 μm in diameter × 0.25 μm film thickness). Pure He was used as the carrier gas at a constant flow rate of 1 mL min^−1^. Furthermore, 1 μL of the extract was injected in splitless mode, with ionization of the sample components performed at 70 eV. The oven temperature was initially maintained at 60 °C, then increased at 10 °C min^−1^ to a final temperature of 280 °C over 30 min. Mass spectra were recorded in the m/z range of 45–550 at a scan rate of two scans per second. The total GC runtime was 52 min. Compound identification was performed by comparing the mass spectra with those in the NIST05 Mass Spectral Library.

### 4.9. Statistical Analysis of Antioxidant Activity and Literature Comparison

T-tests were used to determine differences in antioxidant activity (measured by ABTS and DPPH assays) and to compare with the data reported in the literature. The results were considered statistically significant at *p* < 0.05. Statistical analyses were performed using Statgraphics Centurion XVIII software, version 18.1.16 (Statgraphics Technologies, The Plains, VA, USA).

## 5. Conclusions

An in-depth characterization of the active compounds in Panamanian *C. cajan* leaves was conducted, exhibiting their potential for various applications. Using ethanolic extraction assisted by ultrasonication, phytochemical analysis, and visible spectrophotometry, significant secondary metabolites such as alkaloids, tannins, saponins, and steroids were identified. Remarkably, high levels of phenolic and flavonoid contents were observed, contributing to the exceptional antioxidant capacity of the leaf extract.

Furthermore, GC-MS analysis revealed 31 bioactive compounds, many of which have never been reported in *C. cajan* leaves before. These identified compounds demonstrated multiple promising biological activities, including antimicrobial, antitumor, anti-inflammatory, and antioxidant effects. These properties underscore *C. cajan*’s potential as a source of bioactive molecules for developing novel medicinal and therapeutic products.

The unique environmental conditions of Panama, including its climate, soil composition, and geographical location, may have contributed to the distinct phytochemical profile observed in this study. These geographic and ecological factors can influence the production of bioactive compounds, further emphasizing the importance of investigating *C. cajan* across different regions. These findings not only expand the knowledge of the nutraceutical and ethnobotanical properties of *C. cajan* but also identify potential biotechnological applications that could strengthen the agricultural sector in Panama.

Therefore, a solid foundation is established for future research into the pharmacological, agricultural, and industrial potential of *C. cajan*, encouraging its broader use in health applications and agronomy. Further investigation into the bioactive compounds of *C. cajan*, particularly in relation to the environmental factors of different geographical regions, will be essential for fully harnessing its potential across various industries, positioning the plant as a vital resource for sustainable innovation.

## Figures and Tables

**Table 1:** Active compounds identified in the mass spectra of *C. cajan* leaf extracts.

Compounds	Compounds Type	Mol. Formula	Tr (Min)	Peak Area (%)	Mol. Wt. (g mol^−1^)	Top Peak	Frag. Ions (*m*/*z*) 2nd and 3rd Highest	Extract
α-Guaiene	Sesquiterpenes	C_15_H_24_	10.41	0.2	204	105	147 and 107	Crude
(−)-α-Himachalene	Sesquiterpenes	C_15_H_24_	10.61	0.2	204	189	119 and 105	Crude
cis-(−)-2,4a,5,6,9a-Hexahydro-3,5,5,9-tetramethyl(1H) benzocycloheptene	Aromatic hydrocarbon	C_15_H_24_	10.96	0.1	204	93	133 and 105	Crude
α -Selinene	Sesquiterpenes	C_15_H_24_	11.17	0.1	204	189	93 and 204	Crude
Phytol	Acyclic diterpene alcohol	C_20_H_40_O	15.27	0.9	296	82	81 and 95	Crude
Hexadecanoic acid	Phenolic acid, saturated fatty acid	C_16_H_32_O_2_	16.09	1.4 and 4.1	256	73	60 and 55	Crude and LLE with hexane
Ethyl palmitate	Fatty acid ethyl ester	C_18_H_36_O_2_	16.37	0.2, 13.3 and 5.9	285	88	101 and 55	Crude and LLE with EtOAc and hexane
(E,7R,11R)-Phytol	Diterpenoid	C_20_H_40_O	17.53	0.5	296	71	123 and 57	Crude and LLE with EtOAc
Linolenyl alcohol	Fatty primary alcohol	C_18_H_30_O_2_	17.80	0.5	278	79	55 and 95	Crude
Ethyl linolenate	Fatty acid ethyl ester	C_20_H_34_O_2_	18.02	0.2 and 8.1	306	79	67 and 95	Crude and LLE with EtOAc
Ethyl stearate	Fatty acid ethyl ester	C_20_H_40_O_2_	18.22	0.1, 6.8 and 5.2	312	88	101 and 55	Crude and LLE with EtOAc and hexane
1-Piperidineacetonitrile,. alpha. -styryl	Heterocyclic	C_15_H_18_N_2_	19.39	9.2	226	226	225 and 165	Crude
Pinostrobin chalcone	Chalcones	C_16_H_14_O_4_	20.19	10.9	270	270	193 and 166	Crude
4-(4-Methoxyphenyl)-6,7-dimethoxy-1,2,3,4-tetrahydroisoquinoline	Tetrahydroisoquinolines	C_29_H_50_O_2_	20.90	0.8	299	98	239 and 57	Crude
Squalene	Triterpenoid	C_30_H_50_	23.37	1.4, 2.8 and 1.04	410	69	81 and 95	Crude and LLE with EtOAc and hexane
γ-Tocopherol/Vitamin E	Tocopherol	C_28_H_48_O_2_	26.00	0.8 and 2.0	416	416	151 and 417	Crude and LLE with hexane
n-Heptadecane	Alkane	C_17_H_36_	26.44	0.2	240	57	71 and 85	Crude
Vitamin E	Tocopherol	C_29_H_50_O_2_	27.22	1.8	431	430	165 and 432	Crude
β-Amyrin	Pentacyclic triterpenoid	C_30_H_50_O	31.00	3.5 and 0.7	426	218	203 and 219	Crude and LLE with EtOAc
Lup-20(29)-en-3-one	Triterpenoid	C_30_H_48_O	32.16	12.7 and 21.0	425	205	109 and 424	Crude and LLE with EtOAc
Lupeol	Pentacyclic triterpenoid	C_30_H_50_O	32.77	3.4 and 6.6	426	189	218 and 207	Crude and LLE with EtOAc
2,4-Di-tert-butylphenol	Phenol	C_14_H_22_O	11.24	1.3 and 2.9	206	191	206 and 57	LLE with EtOAc and hexane
Di-isobutyl phthalate	Phthalate ester	C_16_H_22_O_4_	15.20	0.4	278	149	223 and 57	LLE with EtOAc
Methyl palmitate	Fatty acid methyl ester	C_17_H_34_O_2_	15.73	1.8 and 1.6	270	74	87 and 55	LLE with EtOAc and hexane
Ethyl 9-hexadecenoate	Fatty acid ester	C_18_H_34_O_2_	16.30	1.0	282	55	88 and 96	LLE with EtOAc
8,11-Octadecadienoic acid, methyl ester	Fatty acid methyl ester	C_19_H_34_O_2_	17.33	0.8	294	67	81 and 55	LLE with EtOAc
cis-11,14,17-Eicosatrienoic acid methyl ester	Fatty acid methyl ester	C_21_H_36_O_2_	17.40	1.0	320	79	67and 95	LLE with EtOAc
Methyl isostearate	Ester	C_19_H_38_O_2_	17.60	0.7 and 0.5	298	74	87 and 298	LLE with EtOAc and hexane
Ethyl linoleate	Fatty acid ethyl ester	C_20_H_36_O_2_	17.93	4.4 and 1.9	308	67	81 and 95	LLE with EtOAc and hexane
Oleamide	Fatty amide	C_18_H_35_NO	19.72	7.8 and 13	218	59	72 and 55	LLE with EtOAc and hexane
9,12-Octadecadienoic acid, ethyl ester	Fatty acid ethyl esters	C_19_H_38_O_4_	20.85	2.9	330	98	239 ad 57	LLE with EtOAc
Monoethylhexyl phthalic acid	Mono(2-ethylhexyl) ester of benzene-1,2-dicarboxylic acid	C_16_H_22_O_4_	21.10	2.4	278	149	167 and 57	LLE with EtOAc
β-amyrone	Pentacyclic triterpenes	C_30_H_48_O	31.05	4.2	424	218	203 and 219	LLE with EtOAc
Levoglucosenone	Anhydrohexose and a deoxyketohexose	C_6_H_6_O_3_	6.15	1.4	126	98	96 and 68	LLE with hexane
Benzene, 1,3-bis(1,1-dimethylethyl)-	Phenylpropanes	C_14_H_22_	7.90	0.2	190	175	57 and 90	LLE with hexane
1-Pentadecene	Alkene	C_15_H_30_	9.68	0.4	210	55	83 and 69	LLE with hexane
Pyrogallol	Phenolic	C_6_H_6_O_3_	9.91	6.4	126	126	52 and 80	LLE with hexane
1-Nonadecene	Alkene	C_19_H_38_	12.10	0.3	266	83	55 and 97	LLE with hexane
Di-isobutyl phthalate	Phthalate ester	C16H22O4	15.20	1.1	278	149	57 and 150	LLE with hexane
Dibutyl phthalate	Phthalate ester	C_16_H_22_O_4_	16.15	2.8	278	149	150 and 205	LLE with hexane
8-Octadecenoic acid, methyl ester	Oleic acid methyl ester	C_19_H_36_O_2_	17.38	0.6	296	55	74 and 69	LLE with hexane
Elaidic acid	Trans-isomer of oleic acid	C_18_H_34_O_2_	17.77	2.4	282	55	69 and 83	LLE with hexane
Ethyl oleate	Fatty acid ethyl ester	C_20_H_38_O_2_	18.00	4.9	310	55	69 and 83	LLE with hexane
Dioctyl phthalate	Phthalate ester	C_24_H_38_O_4_	21.15	1.4	390	149	167 and 279	LLE with hexane

Note: The abbreviations used in the table are as follows: Mol. formula (molecular formula of the compound), Tr (min) (retention time in minutes), Mol. wt. (molecular weight), Top peak (main peak identified in the mass spectrum), and Frag. ions (*m*/*z*) (fragment ions, indicated as mass-to-charge ratio in the mass spectrum).

**Table 2:** Active compounds identified in *C. cajan* leaf extracts and their reported properties and applications.

Compound	Properties/Use	References
α-Guaiene	Precursor to rotundone (peppery aroma and flavor). Possesses antimicrobial activity.	[26,27]
(−)-α-Himachalene	Antimicrobial agent	[28]
α-selinene	Anti-plasmodial and anti-inflammatory biomarkers.	[29,30]
Phytol	Precursor of vitamin E and vitamin K1. Modulates transcription in cells via transcription factors PPAR-alpha and retinoid X receptor (RXR). Flavoring agent. Antimicrobial, cytotoxic, antitumor, antimutagenic, anti-teratogenic, antibiotic-chemotherapeutic, antidiabetic, lipid lowering, antispasmodic, anticonvulsant, antinociceptive, antioxidant, anti-inflammatory, anxiolytic, antidepressant, immunoadjuvancy, hair growth facilitator, hair fall defense and antidandruff activities. Inhibits osteoclast differentiation.	[31,32,33,34]
Hexadecanoic acid	Anti-inflammatory agent	[35]
Ethyl palmitate	Antiviral agent	[36]
Linolenyl alcohol	Antibacterial and anticancer agent	[37,38]
Ethyl linolenate	Antinociceptive activity	[39]
β-amyrone	Anti-inflammatory and anti-obesity agent	[40,41]
Ethyl stearate	Protective effect against the neurotoxin 6-hydroxydopamine	[42]
Squalene	Antitumor, immunity enhancement, antioxidant, detoxifier, skin senility resistance, hypolipidemic, and antibacterial activities.	[43]
γ-tocopherol	Antioxidant, anti-inflammatory, cancer prevention, and contributions to natriuresis.	[44,45,46]
Heptadecane	Potential biofuel precursor	[47,48]
D-α-Tocopherol	Antioxidant and modulates lipid peroxidation.	[44,49,50]
β-Amyrin	Antimicrobial activity and protects against Alzheimer’s disease.	[51,52]
Lupenone	Innovative drug for preventing and treating diabetic nephropathy. Anti-inflammatory agent	[53,54]
Lupeol	Anti-inflammatory, antiapoptotic, anticancer, antioxidant, and antimicrobial activities. Protective effects on cardiovascular diseases.	[55,56]
2,4-Di-tert-butylphenol	Antitubercular, anticancer, antioxidant, anti-inflammatory, insecticidal, herbicidal, and antimicrobial activity. Exhibits broad toxicity in human and animal cells.	[57,58,59,60]
Di-isobutyl phthalate	Human health risks, including fetal toxicity.	[61,62]
Methyl palmitate	Neuroprotective and cardioprotective activity.	[63,64]
cis-11,14,17-Eicosatrienoic acid methyl ester	Antifouling activity.	[65]
Methyl isostearate	Skin conditioning agent Emulsifier	[66]
Ethyl Linoleate	Insecticidal, antibacterial, and anti-inflammatory activity.	[67,68]
Oleamide	Neuroprotective effects.	[69]
Levoglucosenone	Bio-based platform for drug discovery.	[70]
1,3-ditert-butylbenzene	Mosquitocidal activity.	[71]
1-Pentadecene	Insect repellent.	[72]
Pyrogallol	Antibacterial and antifungal agent.	[73,74]
1-Nonadecene	Anti-inflammatory activity	[60]
Di-isobutyl phthalate	Plasticizer and anti-androgenic effects.	[75]
Dibutyl phthalate	Biosensors, plasticizers, and endocrine disruptor.	[76,77,78]
Elaidic acid	Boosts tumoral antigen presentation and cancer immunity. Treatment of colorectal cancer.	[79,80]
Ethyl oleate	Promotes the drying of fruits, vegetables, and grains Effective microemulsion ingredient to increase the bioavailability of a drug Gasoline additive	[81]
Dioctyl phthalate	Plasticizer	[82,83,84]

## Data Availability

Data are contained within the article.

## Abbreviations

The following abbreviations are used in this manuscript:

ABTS	2,2′-azino-bis(3-ethylbenzothiazoline-6-sulfonic acid)
ABTS+	ABTS radical cation
DPPH	2,2-diphenyl-1-picrylhydrazyl
GC-MS	Gas chromatography–mass spectrometry
FT-IR	Fourier transform infrared
TPC	Total phenolic content
GAE	Gallic acid equivalents
TFC	Total flavonoid content
QE	Quercetin equivalents
TAC	Total anthocyanin content
TEAC	Trolox equivalent antioxidant capacity
TE	Trolox equivalents
LLE	Liquid–liquid extraction
EtOAc	Ethyl acetate
SET	Single-electron transfer
HPLC	High-performance liquid chromatography
ATR	Attenuated total reflectance
